# Supplementary figures and images for: A STIM dependent dopamine-neuropeptide axis maintains the larval drive to feed and grow in Drosophila
Source: PLoS Genet. 2023 Jun 26;19(6):e1010435. doi: 10.1371/journal.pgen.1010435 (PMC10328320; doi:10.1371/journal.pgen.1010435)

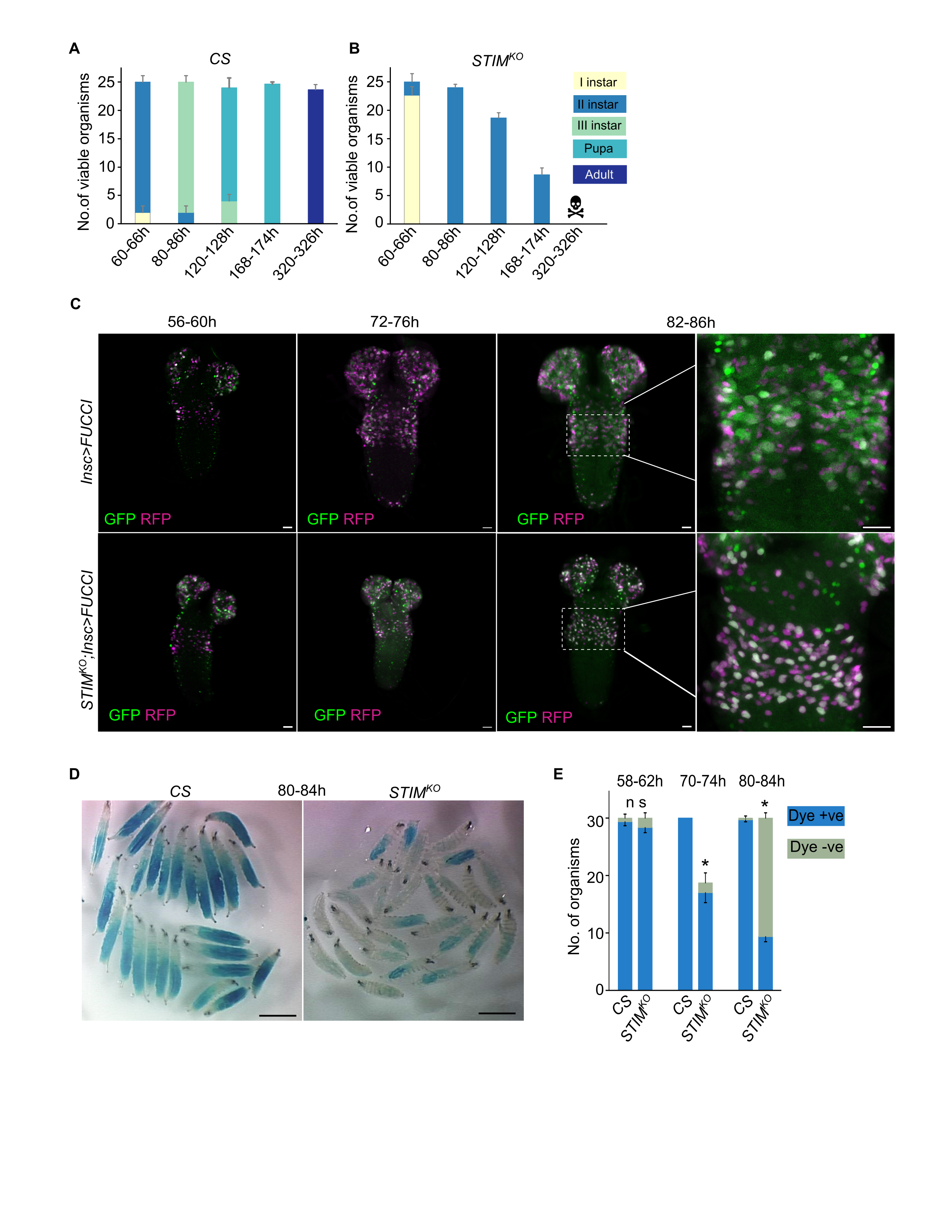

Supplement: S1 Fig — (A-B). Number of viable organisms (mean ± SEM) at the indicated developmental stage of CS and STIMKO after egg laying. Number of sets (N) = 3, number of organisms per set (n) = 25. (C). Confocal images of larval brains at the indicated time points expressing the FUCCI marker. Control genotype is Insc>FUCCI (top row) and the mutant genotype is STIMKO; Insc>FUCCI (bottom row). Here late mitosis/G1 phase, S-phase and G2/early mitosis are marked by green, red, and yellow fluorescent indicators respectively. Scale bar = 20mm; n = 5 larval brains. (D). Representative image of dye-fed larvae from CS and STIMKO at 80-84h AEL. Scale bar: 2mm. (E). Bar graph showing the average number of Dye+ve (presence of blue dye in the gut) and Dye-ve (absence of blue dye in the gut) CS and STIMKO larvae at the indicated ages (mean ± SEM). Number of feeding plates per time point (N) = 3, number of larvae per plate (n) = 30. *P < 0.05, Student’s t-test with unequal variances. P values are given in S2 Table. (TIF) [file pgen.1010435.s001.tif]

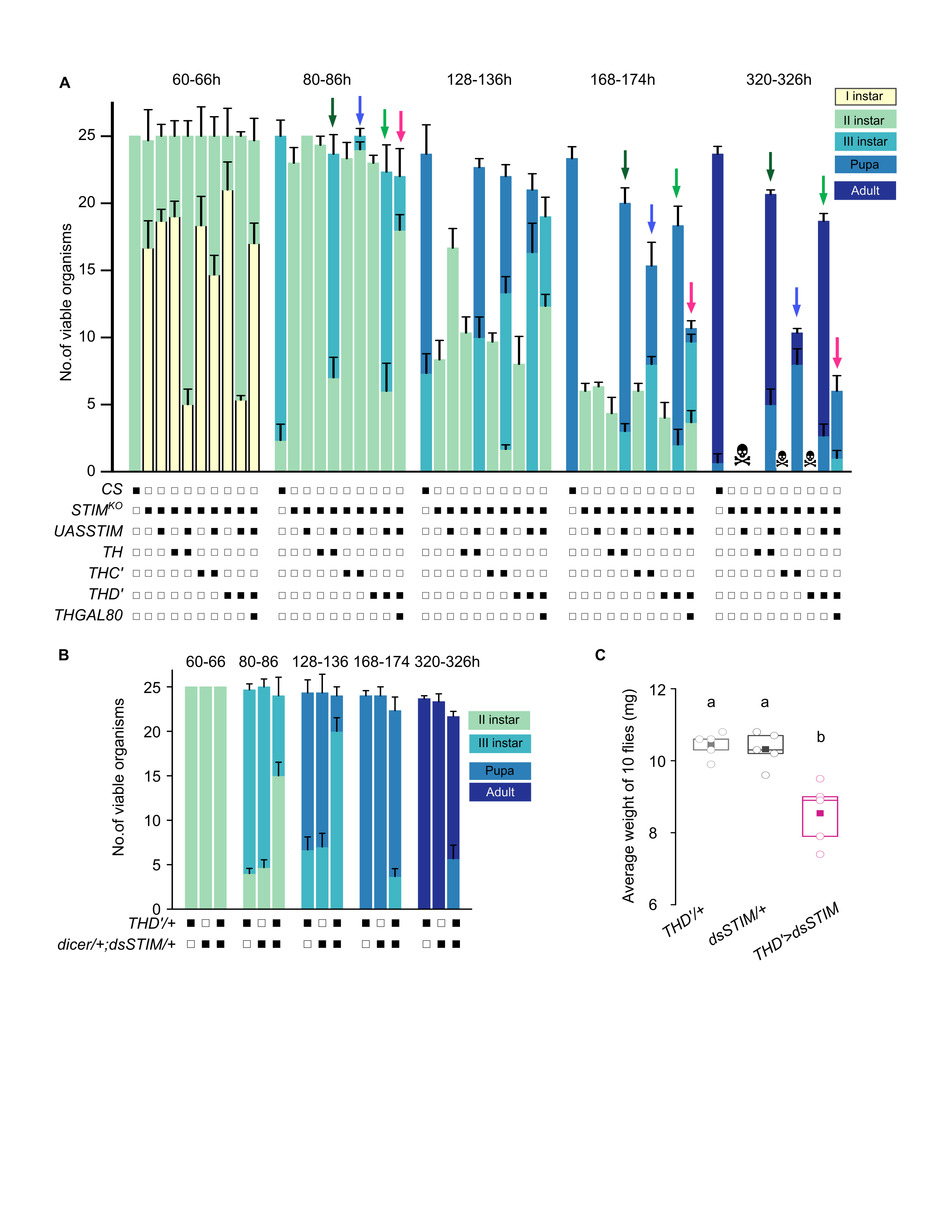

Supplement: S2 Fig — (A). Stack bar graph showing the number of viable organisms (mean ± SEM) and their developmental stage at the specified hours after egg laying for the indicated genotypes. Coloured arrows mark bars that exhibit rescue of STIMKO upon expression of STIM+ driven by THGAL4 (dark green), THC’GAL4 (blue), THD’GAL4 (green) and restricted rescue in presence of THGAL80 (red) with all appropriate genetic controls as indicated. Number of sets (N) = 3, number of organisms per set (n) = 25. (B). Stack bar graph showing the number of viable organisms (mean ± SEM) and their developmental stage at specified hours after egg laying for the indicated genotypes; THD’/+, dicer;+;dsSTIM/+ (controls) and THD’>dicer,dsSTIM. Number of sets (N) = 3, number of organisms per set (n) = 25. (C). Quantification of weight of 10 flies from indicated genotypes. Box plots show the 25th and 75th percentiles with median (bar), mean (square) and each circle represents one set. Each set consists 10 flies of which 5 are females and 5 are male adult flies collected at 6-8h after eclosion. Number of set (N) ≥5. Alphabets indicate different statistical groups. In all panels significant changes between relevant genotypes at the indicated stages were calculated by one way ANOVA followed by post-hoc Tukey’s test. P values are given in S2 Table. (TIF) [file pgen.1010435.s002.tif]

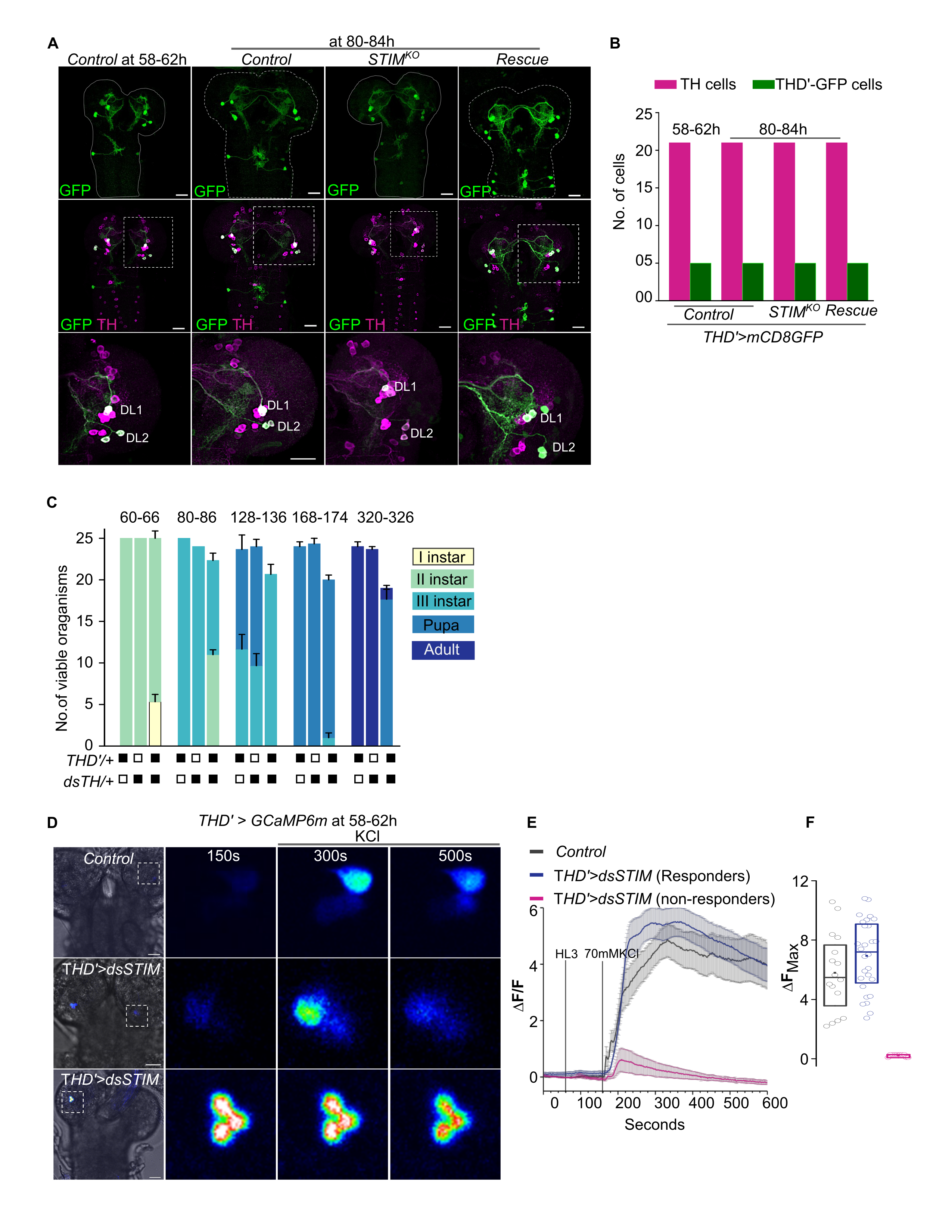

Supplement: S3 Fig — (A). Representative confocal images of larval brains showing THD’ neurons marked with anti-GFP (green) and anti-TH (red) from control (THD’>mCD8GFP), STIMKO (STIMKO; THD’>mCD8GFP) and rescue (STIMKO;THD’>mCD8GFP, STIM+) animals at the indicated developmental time points. Scale bars = 20μm. (B). Numbers of TH positive cells (magenta) and THD’ cells (green) in the larval CNS of the indicated genotypes. Cells were quantified from THD’>mCD8GFP (control) at 58-62h and 80-84h and from STIMKO;THD’>mCD8GFP (STIMKO) and STIMKO;THD’>mCD8GFP, STIM+ (rescue) at 80-84h. The numbers of TH+ve and GFP+ve cells were counted manually and were no different among four hemi-lobes from four brains of a single genotype and among all brain hemi-lobes of all genotypes. Hence the absence of error bars. (C). Stack bar graph showing the number of viable organisms (mean ± SEM) and their developmental stage at specified hours after egg laying for the indicated genotypes THD’/+, dsTH/+ (controls), and THD’>dsTH. Number of sets (N) = 3, number of organisms per set (n) = 25. Significant changes were calculated by one way ANOVA followed by post-hoc Tukey’s test. P values are given in S2 Table. (D). Representative images of the central brain (left panels) indicating the region of focus (boxed), followed by images of THD’ cells with Ca2+ transients at the indicated time points after addition of a depolarizing agent (KCl, 70mM). Ca2+ transients were measured in the indicated genotypes: THD’>GCaMP6m and THD’>dicer;dsSTIM,GCaMP6m by measuring changes in the intensity of GCaMP6m fluorescence. Scale bar = 20μm. (E). Quantified changes in GCaMP6m fluorescence (mean ± SEM of ΔF/F) from THD’ neurons of the indicated genotypes. Number of brains, (N) ≥ 6, number of cells, (n) ≥11. (F). Peak intensities of GCaMP6m fluorescence (ΔF) in THD’ cells from the indicated genotypes. Box plots show 25th and 75th percentiles, the median (bar), and mean (square) of ΔF of each cell (small circles). (TIF) [file pgen.1010435.s003.tif]

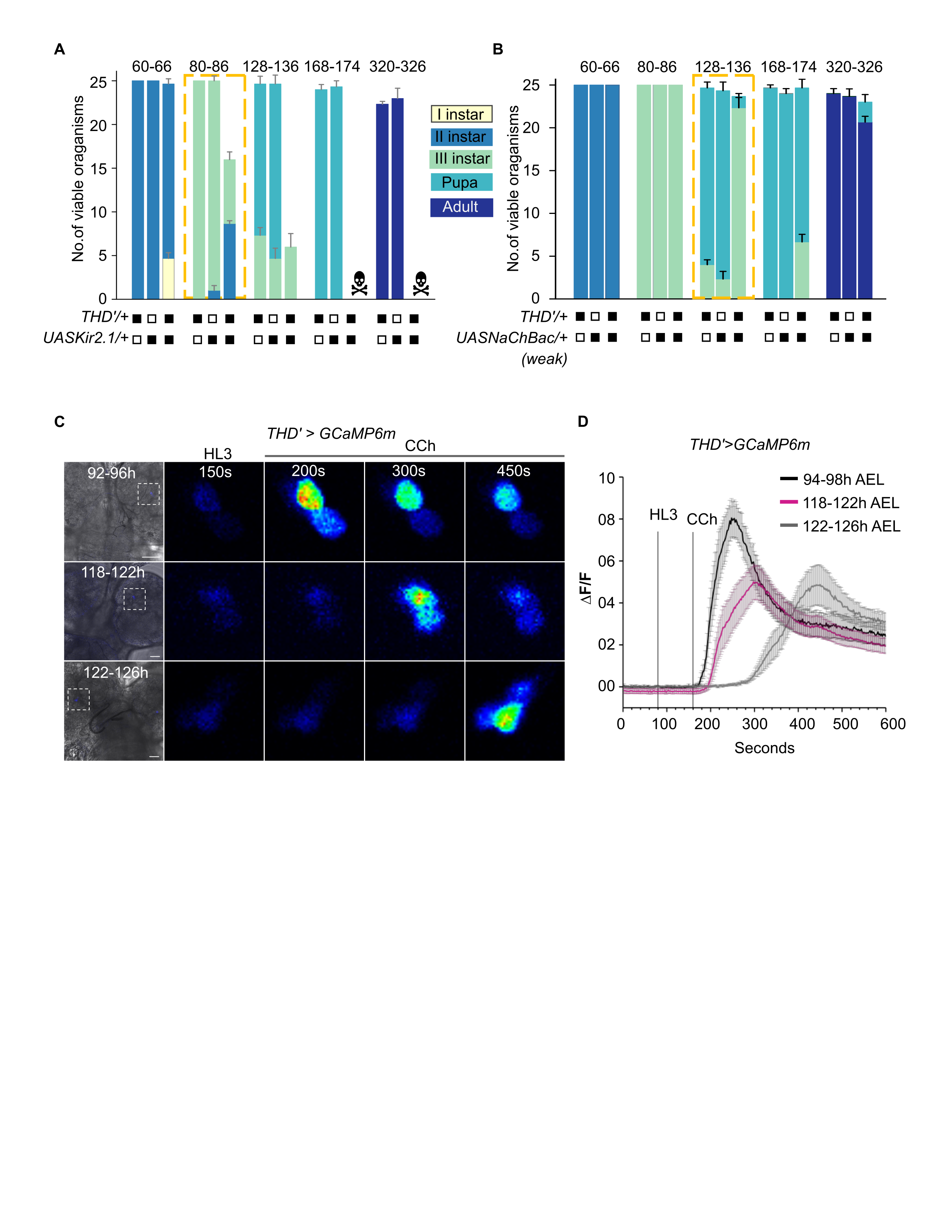

Supplement: S4 Fig — (A-B). Altered excitability in THD’ neurons by expression of either a mammalian inward rectifying potassium channel (Kir2.1) or a bacterial sodium channel (NachBac) affects larval developmental progression and viability. Stack bar graphs show the number of viable organisms (mean ± SEM) and their developmental stage at the indicated hours after egg laying (top) for the indicated genotypes. Number of sets (N) = 3, number of organisms per set (n) = 25. P values calculated after one way ANOVA followed by post-hoc Tukey’s test are given for relevant stages and genotypes in S2 Table. (C). Representative images of the central brain (left panels) indicating the region of focus (boxed), followed by images of THD’ cells with Ca2+ transients at the indicated time points after addition of mAChR agonist (CCh 100μM). Ca2+ transients were measured in the indicated genotype by measuring the changes in the intensity of GCaMP6m fluorescence. Scale bar = 20μm. (D). Quantified changes in GCaMP6m fluorescence (mean ± SEM of ΔF/F) from THD’ neurons of the indicated genotypes. Number of brains, (N) ≥ 4, number of cells, (n) ≥12. (TIF) [file pgen.1010435.s004.tif]

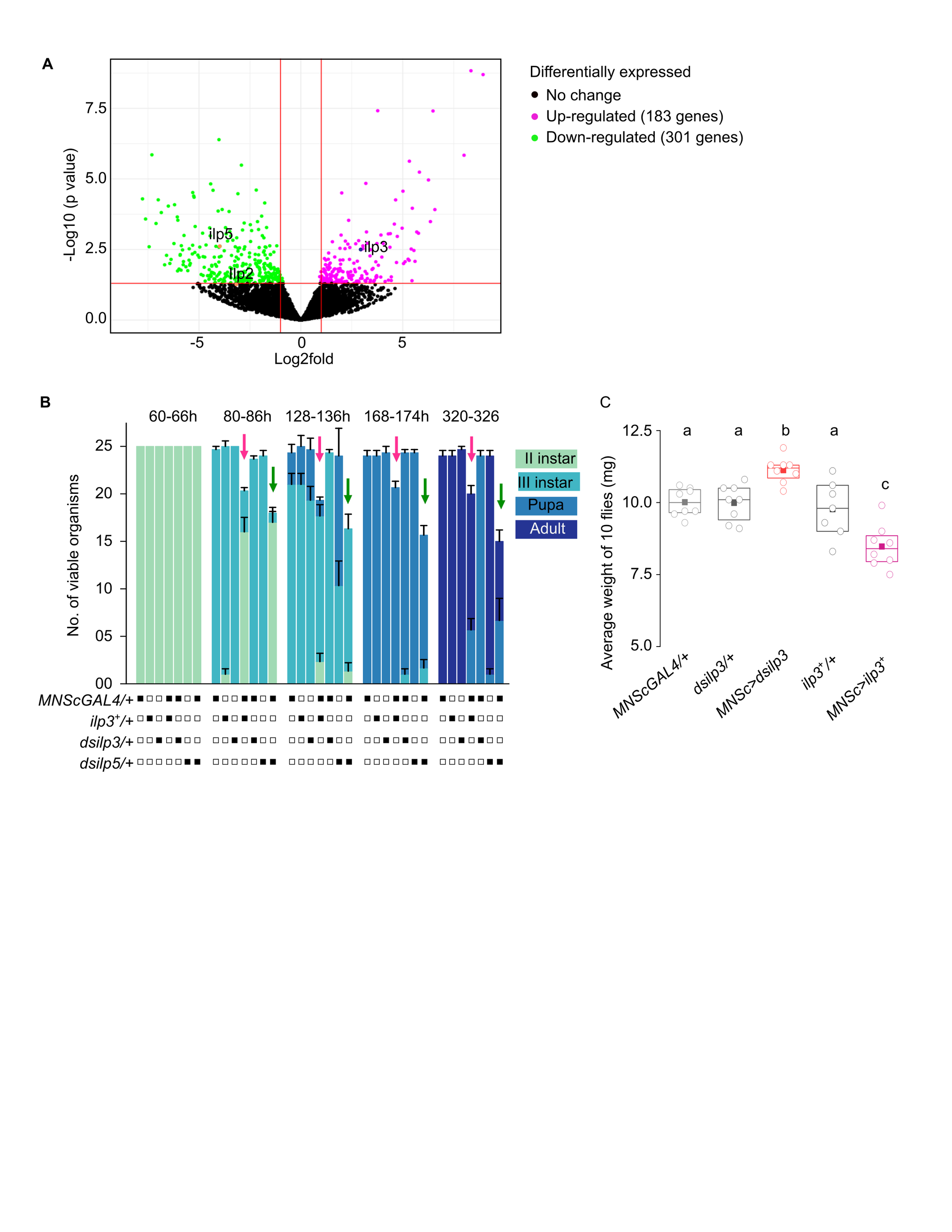

Supplement: S5 Fig — (A). Upregulated (magenta colour, log2fold ≥ +1; p<0.05) and downregulated (green, log2fold ≤ -1; p<0.05) gene sets in STIMKO larval brains (72-76h AEL) as compared to CS larval brains (58-62h AEL), depicted as a volcano plot. N = 4. Also see S3 Table for gene names and their mean expression levels. (B). Larval developmental profile with overexpression of ilp3 in MNSc (red arrow) appears similar to knockdown of ilp5 (green arrow). Stack bar graph showing the number of viable organisms and their developmental stage at the specified hours after egg laying for the indicated genotypes. N = 3 sets with 25 larvae in each set. (C). Knockdown and overexpression of ilp3 in the MNSc affects adult weights in opposing directions. Quantification of weight of 10 flies from the indicated genotypes. Box plot shows the 25th and 75th percentiles with median (bar), mean (square). Each circle represents one set consisting of 10 flies in each of which 5 females and 5 males were collected at 6-8h post- eclosion. A minimum of 5 sets were quantified for each genotype. Alphabets indicate statistically different groups based on P values calculated after one way ANOVA followed by post-hoc Tukey’s test, given for relevant stages and genotypes in S2 Table. (TIF) [file pgen.1010435.s005.tif]
