# Supplementary material for: A STIM dependent dopamine-neuropeptide axis maintains the larval drive to feed and grow in Drosophila
Source: PLoS Genet. 2023 Jun 26;19(6):e1010435. doi: 10.1371/journal.pgen.1010435 (PMC10328320; doi:10.1371/journal.pgen.1010435)
Supplement: Attachment — Submitted filename: Response to reviewers.docx [file pgen.1010435.s017.docx]

Dear Greg,

We are now submitting a revised version of our manuscript where we have included several new experiments to address fully the exhaustive and careful comments of the three reviewers. In addition, as suggested we have re-written certain sections and in part the introduction to focus on larval feeding behaviour. Our point-by-point responses to each reviewer are given below:

**Reviewer #1:** Like other juvenile organisms, food intake, the quality and quantity of food ingested, can influence Drosophila melanogaster juvenile size. This study describes a subset of dopaminergic neurons marked by a THD-Gal4 that appears to maintain the larval motivation to feed. Dopamine release from these neurons requires the ER Ca2+ sensor STIM. Early larvae with loss of STIM stop feeding, grow and eventually die. The authors show that the expression of STIM in the THD neurons rescues the feeding, growth and viability of STIM null mutants. STIM maintains excitability and can partially rescue the dopamine release from THD neurons. The authors used optogenetic stimulation of THD neurons to identify connectivity to neuropeptidergic cells and found that loss of STIM in THD cells alters the developmental profile of specific insulin-like peptides, downregulating Ilp2 and Ilp5, and upregulating Ilp3. Silencing Ilp3 partially rescues STIM null mutants, and strikingly overexpression of ilp3 in larvae affects development and negatively growth. Together these studies identify a STIM-dependent dopamine-ILP circuit that regulates developmental changes in larval feeding behaviour.


The data are very interesting and intriguing; the figures are well-constructed, solid, and well-presented. Unfortunately, there are important problems to be clarified about the mechanism, which is still obscure. There is a lack of experiments that address the problem directly. The overall data suggest that development and growth are arrested first, and then feeding is finalised. The authors suggest that feeding is the main phenotype, but they do not show that STIM or Dopamine has an instructive role, and they do not show clear data that integrate feeding with growth in a direct way. The poor rescue of dsIlp3 raises important questions, particularly given the strong genetic background of transgenes. The rescue of NaChBac or the role of Dopamine release are also poor or unclear. The introduction needs some work and focus on the juvenile stage. Key publications are missing, and others that are not relevant are discussed at length.

*Ans: We thank the reviewer for the exhaustive review. As suggested we have revised the introduction to focus on the juvenile stage. References to adult feeding have been removed, references to larval feeding emphasised and several new references, including more recent ones, added. Results from new experiments to address the role of ilp3 and dopamine release have been added for which the details are given below.*

Major comments:
Given the great differences in the control of feeding behaviour between larvae and adult flies, the citations of articles and circuits, sometimes related to adults and sometimes to larvae, and sometimes without clarifying if they refer to larvae or adults, are confusing, giving rise to possible misinterpretations. Authors need to rewrite and restructure their introduction and also avoid over-interpretations of published articles. Next, I comment on cited articles in the introduction, which need to be fixed or toned down.

Line 49-50. It seems reasonable that the appetite is regulated generally depending on the nutritional status. However, these two articles they cite to support the claim that increased appetite is associated with accelerated growth are articles that show genetic manipulations or sensing of the internal state occurring "despite normal feeding behaviour" (PMID: 14505573). These nutrient-sensing pathways inhibit larval feeding, further showing that appetite, sensing internal states, and growth can be genetically separate. Along the same lines, the work of Layalle and Leopold (PMID: 18854141) deals with the intrinsic mechanisms that control larval growth and puparium time.

*Ans: This section has been re-written with appropriate references. Reference of Colombani et al (PMID:14505573) is now in the discussion (58, line number 490), whereas PMID 18854141 is deleted.*

Wu et al. show that NPF neural system is not an essential part of the basal feeding machinery but is crucial for normal food response under deprived circumstances (motivational feeding). Silencing by RNAi "did not decrease the size and weight compared with wild-type flies". Therefore the statement in the current manuscript that "Mutants for short Neuropeptide F (sNPF) affect body size by regulating food intake [14]" seems incorrect.

*Ans: NPF and sNPF are two different neuropeptides that BOTH affect larval feeding in different ways. We have re-written this section to clarify their separate effects (lines 67-73).*

Line 54-55: "Allatostatin A (Asta), Drosulfakinin DSK) and Tackykinin (TK) access the internal metabolic state and terminate feeding based on the energy state of the organism [8–11]". These circuits relate to adult flies; I think they do not control larval growth. Martelli et al. are also regulation of feeding in adult flies. SIFamide is adult feeding behaviour.

*Ans: This section of the introduction has been re-written and most references to adult feeding have been deleted.*

Reference 15, Schoofs A, Hückesfeld S, Pankratz MJ relates to ex vivo studies of feeding motor neurons.

*Ans: This paper describes serotonergic modulation of gut motility related to feeding in larvae. Hence it has been retained in the introduction (line 79-80).*

Reference 16, Youn H, Kirkhart C, et al. relates to octopaminergic neurons that promote feeding initiation in adult Drosophila melanogaster.

*Ans: This reference has been deleted.*

References 22 and 23, refer to modulations but not to stop feeding.

*Ans: Reference 22 has been deleted. However we have retained reference 23 (19, line 96) which describes behaviour related to cessation of feeding in third instar larvae.*

The idea that what stops feeding is the animal's nutritional status is interesting, but not supported by published data (yet). The current view is that larval size determines the time of feeding cessation and food exit (Stern and Emlen, 1999) and is mechanistically related to an ecdysone pulse. Therefore, the statement: "Multiple neuronal circuits thus integrate sensory inputs and internal metabolic state to maintain feeding and appropriate feeding behaviour in larvae and adults" should be toned down.

*Ans: We have removed this sentence from the introduction. It is likely that changes in ILPs affect ecdysone release – this aspect has been mentioned in the introduction (line 89-91).*

The authors should avoid over and misinterpretations of papers, perhaps eliminating the adult papers or clearly defining when they refer to feeding regulation during growth or in adult animals.

*Ans: We have removed most references to adult feeding behaviour.*

Figure 1H. The size and feeding rate of STIM-KO mutants are similar to early 60-72 h AEL larvae that their chronological age, raising the question whether the reduced feeding is because the mutant are arrested at a low feeding stage.
Please quantify the weight or size of the larvae over time. Although it said the images are representative, the images in Figure 1C and 1H show larvae of very different sizes, with the mutant larvae in H much larger than those in C.

*Ans: We have clarified this issue in Fig 1. Larval length has been measured and quantified (Fig 1E). Feeding rates have also been quantified (Fig 1J and 1K). From comparing the two graphs it is evident that feeding is reduced early. We apologise for the previous Figures 1C and 1H where scale bars were placed incorrectly. This is now corrected in panels 1D and 1I. Dye-fed larvae from CS (44h and 60h) and STIM^KO^ (44h, 60h, and 80h) are shown at higher magnification to allow clear visualisation of food in the gut.*

Figure 1I and IJ and Figure 1C suggest than mutant larvae first stop growing and later stop feeding. Please comment. It might be informative to measure larvae after feeding to associated feeding behaviour and growth.

*Ans: Please see panels in the revised figure with larval measurement (1E) and larval feeding rates (1K). It is evident that STIM^KO^ larvae at 36h AEL exhibit reduced feeding.*

Figure 2 rescue.
STIM rescue in THC'GAL4-labeled neurons is described as poor rescue (5+1 adults; S2A Fig), but rescue by NaChBac or dsIlp3 is even poorer.

*Ans: We agree that rescue from THC’>STIM^+^, THD’ >NachBac and MNSc>dsilp3 are equivalent. The text has been modified to reflect this at lines 201-202, 281-285, 397-406, 484-493 .*

The rescue by STIM overexpression is absolute. Nevertheless, according to Figure 4, which measures Dopamine release, the dynamics of dopamine release do not reach the control level, which questions the importance of dopamine release. It also questions the physiological role of dopamine-insulin in the process, as inferred from the title.

*Ans: It is likely that the observed dopamine release in controls is influenced by other dopaminergic and non-dopaminergic inputs (synaptic or modulatory) that remain mutant for STIM. We have addressed this difference in the discussion in lines 490-496.*

Observing dopamine release, together with the NaChBac data, the THC data, and the poor dopamine receptor defect (only developmental delay), suggests that the underlying (main) defect may be another uncharacterized one. This problem needs to be sorted out.

*Ans: We agree and have addressed this important point by reducing dopamine synthesis in THD’ neurons using the THRNAi. Larvae were tested for feeding and growth (Fig 3 A-D and Sup 3C). The results support our conclusion that dopamine from THD’ neurons is required for feeding and growth.*

Please correct Figure S1 labelling. Confocal (C) and (D) are representative images of dye-fed larvae from CS and STIMKO at 80-84h AEL.

*Ans: Corrected*

According to the authors not all neurons in THD are dopaminergic. Therefore, it should be more correct to refer in Figure 5C. to THD cells rather to dopaminergic neurons, as the contribution of non-TH neurons in the optogenetic experiments is not controlled.

*Ans: Corrected*

Figure S6. The number of adults rescued are n=3 in three experiments of n=10 or

n=30? It seems rather low.

Ans: *This experiment has been repeated 3 times with 25 animals per experimental set (now Figure 6D). Out of 25 larvae, at 320h, 3-4 flies eclosed and within 12hr all remaining pupae (6-8) also eclosed as adults. The rescue is therefore ~40%.*

Given the role of Ilp3 in regulating other Ilps and that Ilp3-KO has no phenotype, the authors should present more data on the possible physiological role of Ilp3 and if feeding is affected or only growth and how an Ilp silencing promotes growth.

*Ans: Our data with knockdown and overexpression of ilp3 are consistent with ilp3 as an anti-growth signal. Knockdown of ilp3 in MNSc leads to larger pupae (Fig 6h and 6I) whereas overexpression leads to smaller larvae ((Fig 6K and 6L). We have included an experiment where we have tested feeding rate of larvae with overexpression of ilp3^+^ in the MNSc. Feeding rate is not affected (Fig 6M). Thus the effect of ilp3 on growth is not through feeding. We have discussed the implications of these data in lines 540-550.*

Figure S1B shows viability, but a curve of changes in larval growth over time would be informative to define the timing of feeding and growth defect.

*Ans: This has been done – please see Fig. 1E and Fig. 1K.*

"The momentum of larval growth is maintained by constant cell division and cell growth. Larval growth occurs by cell growth, and until this moment in the article, the authors had not monitored the cell division of internal organs. Therefore, "Cessation of growth in STIMKO larvae (Fig 1D), suggested a deficit in cell division" seems strange. It would rather suggest a defect in developmental progression or in cell growth.

*Ans: The text has been modified suitably to reflect possible changes in developmental progression or cell growth (lines 136-139).*

Line 127. First, the authors stated that STIM-KO larval neuroblasts existed quiescence as the control brains, but cell division slowed later. Therefore, there is no defect in nutritional input because the "brain sparing" process enables the brain to continue proliferation even in completely starved larvae (once quiescence is released).

*Ans: The brain sparing process of cell growth and cell division occurs in 3^rd^ instar larvae after reaching critical weight (Cheng et al., 2011). Changes in larval and brain size occur much earlier in STIM^KO^ larvae when neuroblast divisions are sensitive to nutrition.*

The impact of slow cell division on neuroblasts and systemic growth is not further discussed. The THD circuit appears to be unaffected, so cell division in these neurons is not affected by reduced feeding? It is, therefore, unclear what the authors attempt to communicate here.

*Ans: As mentioned in lines 135-136 and 161-163 division of neuroblasts is dependent on nutrition (26, 29 and 30) and this is what led us to look at feeding in STIM^KO^ larvae. Cell divisions for larval dopaminergic neurons are complete during embryonic neurogenesis (34****)*** *hence the THD’ circuit is unaffected by loss of nutrition in STIM^KO^ larvae.*

"Based on these results it appears that STIMKO larvae gradually stop feeding from early 2nd instar larvae and the consequent nutritional deficits prevent normal growth."
STIM-KO stops growth or has reduced growth apparent in 60 h AEL, suggesting that slow growth or developmental arrest is an early defect, perhaps even earlier than feeding. The data presented do not clarify what the cause is and what the effect is.

*Ans: Fig 1K shows that feeding is affected early.*

Since the STIM-KO defect is more complex than what has been reported when starving wild larvae of that age, the authors should consider all possibilities and present observations without assuming or over-interpret that one defect is the cause of the another defect. Could feeding L-DOPA rescue the growth or feeding defect?

*Ans: Due to the feeding deficit we have not attempted L-DOPA feeding. Instead as shown in Fig 3 we tested the effect of THRNAi knockdown on feeding and growth.*

Lines 133-135 should be toned down or eliminated.

*Ans: New data shown in Fig 1E and Fig 1K support this sentence.*

Related to THD’>dsSTIM animals. These animals exhibit delayed larval growth and reduced feeding (Fig 2I-2K; S2B Fig) but no larval lethality. It is relevant to present data on the adults, their size, and weight. What is the impact of delayed larval growth and reduced feeding?

*Ans: We have now included these data. In comparison to controls THD’>dsSTIM flies exhibit reduced weight (Sup Fig 2C)*

Line 191: "A few cells (70%) responded…." I think that 70% cannot be described as "a few cells"

*Ans: We apologise - this has been corrected*.

Line 199: The rescue with NaChBac supports the role of STIM-KO in the excitability of THD neurons. However, the explanation of variability due to a stochastic effect of NaChBac is an ad hoc interpretation and an inference of the incomplete effect. It could be that excitability is only part of the phenotype.

*Ans: We agree and have provided alternate interpretations (line 287-289).*

How does STIM control larval growth, feeding and Dopamine release?

*Ans: Our data demonstrate that loss of STIM affects cellular properties of THD’ dopaminergic neurons (such as excitability and ER Ca^2+^ homeostasis) leading to the inability to release dopamine (Discussion 2^nd^ para). We show that 1) dopamine from THD’ neurons is required for larval feeding 2) STIM function in THD’ neurons is required for dopamine release and to maintain appropriate ilp levels for growth. The feeding deficit is presumably through direct dopamine modulation of neuropeptides that regulate feeding (Discussion para 3 and 4).*

Line 228 "Though ER-Ca2+ release, in response to CCh could be measured in just 7 out of 23 cells, the subsequent step of ER-store refilling, presumably after Store-operated Ca2+ entry into the cytosol through the STIM/Orai pathway, could be observed in all control THD' cells… and absence in THD' neurons from STIMKO brains. However, the release of Dopamine is reduced, not absent. The dynamics of STIM-KO, THD>STIM dopamine release suggests additional mechanisms not only defect in dopamine release. Please comment or clarify how to interpret the data in this figure.

*Ans: We have provided a possible interpretation for why dopamine release in rescued animals appears different from wild type in the discussion (lines 490-496).*

Why have authors not tested the potential role of dopamine release more directly?

*Ans: We have done this now by looking at the effect of Dopamine receptor knock downs in MNSc cells and by dopamine stimulation of MNSc>GCaMP6m (Fig 7A-E).*

The effects of dopamine receptors do not clarify whether the main effect of STIM-KO is related to excitability/dopamine release. Line 272. Knockdown of Dop1R1 in peptidergic cells suggests that Dopamine regulates development time, but this datum does not indicate whether there is a control/defect on lfeeding or arval growth deficits that would mimic STIM-KO.

*Ans: In agreement with the data with ilp3 in the revised manuscript we observe growth phenotypes by Dopamine receptor knockdowns in MNSc (Fig 7A-D).*

The experiments with CCh stimulation are interesting but do not add or explain the link between feeding, feeding cessation, and larva growth and defects in STIM-KO animals.

*Ans: The CCh stimulation experiment identifies developmental changes in the response of THD’ neurons that correlate with cessation of feeding. They are thus supportive of a role for THD’ neurons in regulating feeding.*

TransTango in leaky in the larval brain: In the original paper of TransTAngo (https://doi.org/10.1016/j.neuron.2017.10.011) "the sensitivity of trans-Tango (in the larval brain) may lessen in circuits with sparser synaptic contacts. Please comment on this issue, as this is relevant to the validity of the presumed synaptic partners.

*Ans :We agree and have removed the TransTango data from the revised manuscript.*

Lines 300-305. How can Ilp3 silencing rescue larval growth in animals with low levels of Ilp2 and Ilp5? These neuropeptides act through the same receptor. Could InR manipulation rescue STIM-K?

*Ans: A possible explanation for the differential effect of ilp3 could lie in differential affinity for the InR, though to our knowledge this is not documented so far. We have discussed this in lines 544-550. We have not attempted any InR manipulation since these are likely to lead to pleiotropic effects that would be hard to interpret.*

Does ILP3 overexpression in ILP2 neurons reproduce some of the defects of STIM-KO? How Dopamine release inhibits ILP2 and ILP5 and stimulates ILP3?

*Ans: Growth deficits in STIM^KO^ larvae are replicated to some extent by Ilp3 overexpression in MNSc. We see smaller sized larvae (6K and L) and adults with reduced weight (S5E). Overexpression of ilp3 broadly parallels the published effects of ilp5 knockdown, which we replicated as a control (S5C). However, ilp3 overexpression does not lead to a feeding deficit (Fig 6M) indicating that ilp3 acts as an anti-growth signal. We have appropriately modified our conclusions to reflect these data.*

Lines 304-311: These conclusions are overinterpretations. Increased feeding is not the same as extended feeding. The authors must measure the feeding rate of Ilp3 in the STIM-KO animals.

*Ans: We agree. Further experiments indeed show that ilp3 impacts growth but not feeding. The results have been suitably modified.*

"A small proportion of STIMKO larvae rescued by ilp3 knockdown appear significantly larger than control larvae (Fig 6F and 6G), suggesting excess feeding in the absence of ilp3."

This suggestion must be verified with data. If not supported by data, this is a suggestion that Ilp3 may regulate stop feeding.

*Ans: New data of overexpression of ilp3 (Fig6J-M) demonstrate that ilp3 does not affect feeding but it affects growth. This sentence has been modified appropriately (line 412-414).*

Previous work has already shown that brain Ilps can inhibit feeding. However, given that Ilp3 is increased and Ilp2 and Ilp5 are decreased, it is important to assess the net effect. The authors must include the effects of silencing Ilp5, as a control of the effect. Ilp2 silencing is likely lethal.

Ans: *Overexpression of ilp3 and knock down ilp5 both slow down the progression of larval development (Sup Fig 5C) indicating that ilp3 and ilp5 function differently.*

"This idea is further supported by over-expression of ilp3 in MNSc [50] where we observed smaller sized larvae (Fig 6H and 6L), suggesting reduced feeding due to untimely over-expression of ilp3, and a delay in larval moults from 2nd to 3rd instar stage (Fig 6J). Prolonged feeding can be a defect in development timing and ecdysone production or levels, which the authors have not tested.

*Ans: Changes in developmental timing are indeed likely to be due to changes in ecdysone levels that can be modulated by ilps (refs 16 and 17). This aspect of dopamine mediated ilp dysregulation has been included in the discussion (lines 546-549).*

Without testing the impact on feeding rate, the conclusions regarding Ilp3 are overinterpretations: "Taken together, these data show that loss of dopamine signals from THD' cells changes the normal expression profile of ilp3 in the MNCs and demonstrate that in normal development Ilp3 is one of the neuropeptides that signals an end to larval feeding prior to pupariation. Note that Ilp3-KO animals show normal larval growth.

*Ans: This statement has been modified in the revised manuscript and the role of ilp3 in feeding has been modified to a role for ilp3 as anti-growth. Our data demonstrate ilp3 over expression results in smaller larvae (Fig 6K and 6L) and underweight flies (S6E) whereas ilp3 knock down leads to larger pupae (Fig 6H and I) and overweight flies (S6E).*

The discussion of the dopamine-insulin axis in rats is interesting, but is it related to the juvenile stage? Neither mice nor young rats stop feeding when they reach sexual maturity, so it is vague what the established parallelism refers to. The cessation of feeding before metamorphosis is a peculiarity of metamorphosis and is a genetic program. Perhaps the authors should test the STIM-dopamine-insulin axis in adult flies related to adult hunger/satiation.

*Ans: Mice born with a deficiency for dopamine feed poorly and die a few weeks after they are born (64). We agree that studying the dopamine-insulin axis in adult fly feeding would be of interest.*

Reviewer #2: The paper, entitled "A STIM dependent dopamine-insulin axis maintains the larval drive to feed and grow in
Drosophila" describes a new neuronal circuit that regulates larval feeding. Dopaminergic cells in the CNS are essential, which function via the Stromal Interaction Molecule (STIM) on neurosecretory cells that produce the insulin-like peptide ilp3. This circuit is responsible for the age-appropriate regulation of feeding behavior in the Drosophila larva.

Before the paper can be published, I have a few criticisms that the authors should address.

General concerns:

1) The anatomical description of CNS dopaminergic neurons has major gaps. The authors seem to have missed several important publications here. The paper by Selcho et al. 2009 (PlosOne) describes the DL1 and DL2 cluster neurons and the complete larval dopaminergic system in great detail at the single cell level. In addition, even connectome data are available. The DL1 cluster neurons are included in Eichler et al. 2017 (Nature) and Saumweber et al. 2018 (Nature Commun). Here, they are referred to as DAN-c1, DAN-d1, DAN-f1, and DAN-g1. The complete input and output circuits of DL1 dopaminergic neurons were also described at the synaptic level by Eschbach et al. 2021 (Nature Neurosci). A very extensive anatomical description results from this work, which unfortunately has not found its way in here so far. Unfortunately, these studies also strongly suggest that there is no direct synaptic connection between the DL1 cluster and neurosecretory ILP3 cells.
*Ans: We thank the reviewer for pointing out these important papers. We have now read them and included information from them in appropriate places in the text. Selcho et al 2009 (Ref 35), Saumweber et al 2018 (Ref 51) and Eschbach et al., 2020 (ref 50). Based on these we agree that direct synaptic connectivity between the DL1/DL2 clusters and the MNSc is highly unlikely. However our data in Fig 5 and Fig 7 do demonstrate a role for dopaminergic modulation of MNSc activity.*

2) In the above work and other extensive studies from the Gerber, Thum, and Zlatic laboratories, the dopaminergic DL1 neurons are shown to specifically innervate the larval mushroom body and not neurosecretory cells. They are responsible for punishment learning by mediating the negative teaching signal to the mushroom body during olfactory conditioning. This function is also found in adult flies and even in other insects. Important reviews and work on this topic can also be found from the Waddell, Preat, Davis, Rubin laboratories. The work would benefit greatly if these efforts were considered as well.

*Ans: We agree and have included earlier work of DL1/DL2 neurons projecting to the larval MB (see ref 35) and a recent review on feeding circuits in the larval brain (ref 5). Work in adult fly brains may not be of direct relevance and has not been included.*

Specific concerns:

Figure 4A:
The description of the anatomy here is unfortunately incorrect. Based on the single cell description, we already know that the cells from the DL1 cluster innervate the mushroom body. Thus, the middle area shows the innervation of DL1 neurons to the larval mushroom body. The second area is very likely based on basolateral innervation of DL2 neurons. The third area is really incorrectly described here. This area is clearly within the CNS. It clearly overlaps with the NC82 staining in blue. It is an expression in the basomedial CNS that can originate from DL1 and/or DL2 neurons. In addition the labels are messed up and are wrongly described in the figure legend.

*Ans: We thank the reviewer for pointing out these errors and have corrected them in lines 298-305 and the figure legend.*

Figure 5 - Line 251-253:
I'm sorry, but I really disagree here. I see no evidence that the cells of the Transtango sample are identical to that of C929. First, the connectome information for the downstream partners of the DL1 cluster is now available. Based on the synaptic reconstruction (Eschbach et al. 2020) of the DL1 downstream circuit, no neuropeptidergic cells are identified. Second, single-cell analysis of DL2 cells also reveals no inervation of the pars intercerebralis and the neurosecretory cells loacated there. Third, the position of the transtango cells labeled with THD does not overlap with that of the cells labeled with C929. Fourth, when the size of the cells is carefully checked, it is also clear that they are different in size. Fifth, for such a statement, one really needs a co-labeling of the cells of THD-transTango and C929 in one animal. Separate evaluation of two brains from different animals is not acceptable for such a statement. Sixth, the analysis of receptor expression shows (Selcho et al. 2009) that there is no expression of Dop1R in the neurosecretory cells of the pars intercerebralis. Based on these arguments, I unfortunately do not see a synaptic link between dopaminergic and ILP3 cells. Similarly, I lack the data to directly compare C929 Gal4 expression with ILP3 cells.
This will require further analysis showing a clear link. However, the data to date almost exclude such a link.

*Ans; We have now removed all TransTango data which we agree was ambiguous and as mentioned by reviewer 1 shown to be unreliable in larvae. As stated above several independent lines of evidence indicate that MNSc activity is modulated by dopamine release from THD’ cells including knockdown of Dop1R1 in MNSc which results in a development and growth deficit (Fig 5 and Fig 7). Among the MNSc we have not attempted to distinguished between cells that secrete Ilp3, Ilp2 and Ilp5.*

Therefore, I do not believe that the results are evidence for connectivity. In fact, it may not be necessary, since dopamine as a neuromodulator via volume transmission can act over longer distances.
In addition, there is a wonderful description of the anatomy, function, and synaptic connectivity of the larval pars intercerebralis - which you may see here in C929. It is composed of DMS, IPCs, and DH44-positive cells. This work was published by Huckesfeld et al. 2021. Similarly, there are now several publications to obtain specific lines also for neuropeptidergic cells (e.g. Deng et al. 2019, Neuron).

*Ans: We agree and have modified the text accordingly (lines 418-420). The appropriate reference suggested above is included in the revised text (ref 52) among others for volume based transmission (ref 53-56).*

Figure 3A:
This is crucial - are you really sure that you have measured the signal from the same neurons? What if DL1 and DL2 neurons behave differently? There is a lot of data showing that DL1 neurons innervate the mushroom body and transmit reinforcement signals to the mushroom body. Especially in the case of aversive signals. The DL2 neurons, on the other hand, seem to be involved in food intake as you mention in the discussion. So what if the difference in signaling is due to differences between DL1 and DL2? The second row seems to show two DL2 neurons. The first row three DL1 neurons. But what is seen in the third row? Can they show more clearly which cells are involved here? Can't they show all 5 cells? Are there differences between the individual cells which allow to describe the cells individually? Is this maybe also true for the data shown in figure S3D?

*Ans: We have re-analysed these images and find that both DL1 and DL2 clusters respond to KCl. As suggested by the reviewer we have modified figure 3E to include both DL1 and DL2 clusters from two lobes of the same brain. Unfortunately, the two clusters do not come up in the same confocal plane from the same lobe.*

Introduction LIne 51 - 68:
Would it be possible to restructure this part? It reads like a loose enumeration of results. However, it is somewhat confusing because it is rather unstructured and does not clearly distinguish between larval and adult data. It is often not clear whether the results apply to both or only to one developmental stage.

*Ans: This section of the introduction has been re-written and most references to adult feeding have been deleted.*

Figure 1:
The effect is clear - but why do the STIM KO images not focus on the thoracic ganglion? in particular, the last pciture shows both brain hemispheres and not the same part of the thoracic segment as the corresponding control. Also, the second column is displaced and partially encompasses the brain hemispheres.
The figure legend for Figure S1D is missing. The scale bar is also missing for the images.
What does Dye-ve and Dye+ve mean? I found the info in the material and method section, but it is missing in the figure legend.

*Ans: The differences in the images between control and STIM^KO^ are primarily because at 70-74h and 82-86h, STIM^KO^ brains are smaller. Therefore under the same magnification in STIM^KO^ we see both the brain lobes and a part of the ventral ganglia. Figure legend for S1D is added, a scale bar added and an explanation for Dye+ive and -ive included in the legend.*

Line 147 - 150:
I'm not sure if the number of viable adults is a good way for preseting the data. I guess you have to provide at least some ratio or the info that these are 20 out of 25 animals.

*Ans: We have included the information that these numbers are out of a total of 25 animals.*

Line 151: Typo: mCD8-GFP

*Ans: Corrected*

Line 153: Please cite the original data on the anatomy of the larval dopaminergic system (mentioned above).

*Ans: We have changed the references to Selcho et al., 2009 (35); Hartenstien et al., 2017 (34) and Bjordal et al. 2014 (11).*

Figure 2: Please use the labels DL1 and DL2 also in the picture of the brains. It would really help to keep the same descriptions in the text and figures.

Ans: *The DL1 and DL2 clusters have been labelled in Panels 2C and 2D*

Is there also some inter individual variability in the expression of THD-Gal4? It seems that there are only two DL1 neuron and no DL2 neuron on the other side of the brain.

*Ans: We do not see any variability in larval brains for the DL1 and DL2 clusters. The apparent change in numbers between the two sides is due to overlapping cells in the confocal stack. All 5 cells can be distinguished as we move through the Z-planes.*

LIne 175- 176:
How is this calculated? In the supplement it says exactly the ppposite. Why are there no error bars, why are the results all exactly the same? How many brains were really used? Based on the images, I see a lower signal in the STIM KO, especially with two DL2 neurons and one DL1 neuron.

*Ans: We too have observed a lower signal in STIM^KO^ of THD’ driven GFP. On careful observation all DL1 and DL2 cells are present in STIM^KO^ brains albeit with fainter GFP signals – we do not understand the reason for the reduced GFP signal. We have explained how the TH cell numbers have been calculated in the Sup fig 3 legend. Essentially there are no error bars because we did not observe any variability in all four brains from all four genotypes.*

Reviewer #3: In animals, the ability to initiate, maintain, and terminate feeding when nutrients are available is essential for normal development, homeostasis, and fertility. Reduced feeding is associated with a smaller body size and lower fertility, and increased feeding is associated with obesity. Understanding the mechanisms behind the normal regulation of feeding behaviour is therefore an important biological question.

In this manuscript, the authors found that whole-body loss of STIM, an ER calcium sensor, led to a reduction in body size, a severe developmental delay, and the later death of larvae. In particular, the transition from the second to the third instar stage of larval development was impaired. The authors show that animals with whole-body loss of STIM had defects in thoracic neuroblast cell division, and that STIM mutants had reduced food intake compared with control larvae.

To determine the cell type in which STIM might act to mediate these effects the authors partially rescued viability in animals with a functional STIM protein in TH-GAL4-positive neurons, which are dopaminergic. In particular, the brain-specific THD’-GAL4 neurons were mostly responsible for this rescue of viability, and the authors observed improved food intake in these larvae. Importantly, loss of STIM in THD’ neurons delayed larval growth and reduced feeding behavior.

The authors then show that whole-body loss of STIM at later, but not earlier, larval stages caused defects in their THD’ neuron calcium response, which could be rescued by expressing STIM in those neurons. When the authors knocked down STIM in THD’ neurons they found that some animals had a complete failure of calcium responses while others had relatively normal responses, indicating heterogeneity in the neuronal response to STIM loss. The authors partially rescue viability of STIM knockout animals by overexpressing NaChBac in THD’ neurons, and show that STIM is important for dopamine release from the THD’ neurons.

The authors go on to show that the THD’ neurons are functionally connected to peptidergic neurons, with some peptidergic neurons activated by THD’ neuron activity and others inhibited. That dopamine was important for activation of peptidergic neurons was indicated by an experiment in which loss of dopamine receptors on C929-GAL4-positive neurons caused a developmental delay.

The authors show that whole-body loss of STIM caused a decrease in brain levels of ilp2, ilp5 and ilp3, with ilp3 upregulated by more than 5 fold. The authors were able to rescue these altered ilp mRNA levels by THD’-specific expression of STIM, and also partially rescues larval development. In animals with knockdown of ilp3 in the insulin-producing neurons the animals are bigger, and overexpression of ilp3 in these same cells reduced body size.

Based on this data, the authors propose a model in which STIM acts in THD neurons to promote calcium homeostasis and excitability of these neurons downstream of cholinergic inputs. This allows dopamine to be released which binds to Dop1R on the surface of peptidergic neurons, which stimulates (at least in the mNSCs) changes in ilp levels to alter body size.

The underlying idea behind much of this work is very elegant, and some of the findings are highly interesting and suggestive. However, there are multiple methodological inconsistencies, incorrect genotypes, statistical errors, and assumptions that will require a large amount of work to remedy ahead of publication.

Major issues

1. Much of the work describing a cell type-specific role for STIM relies upon rescuing STIM to the THD’ neurons in a whole-body knockout animal. As far as I can tell, in the majority (or all) the cases the control animal is Canton-S (no transgenes), the rescue genotype is STIMKO with THD’ expression of STIM, with STIMKO as the mutant. Unfortunately, the genotypes used by the authors do not support their claim of a cell type-specific role for STIM for the following reasons.
In Drosophila, to show a cell type-specific rescue, it is essential to have the following controls: mutant, GAL4 line in a mutant background, UAS line in a mutant background, UAS+GAL4 in a mutant background. With phenotypes like body size, feeding, and larval development, all of which are affected by a huge number of genes, it is essential to know that neither genetic background “rescues” the phenotype on its own, and that the “leaky” expression of the UAS (which occurs due its insertion site) does not rescue the phenotype either (because then it is not a cell type-specific rescue). Without these proper controls, the authors cannot convincingly show that STIM in the THD’ neurons affects the phenotypes they have shown in the paper. A revised version of the paper must replicate all phenotypes in the paper with the proper controls.

*Ans: We agree that appropriate genetic controls are essential. The complete larval developmental profile of the rescue genotype shown in Figure 2A was first tested with all appropriate controls including STIM^KO^; THD’ and STIM^KO^; UAS STIM. These data are shown in the bar graph in Supplementary figure 2A and this information has now been added to the Figure legend of Fig 2A. Similarly we have performed the appropriate genetic controls for all other genotypes tested and have included them either in the main figure or the associated supplementary figure.*

2. The main message that the authors lay out in the paper is about control of feeding behavior. There are two issues that prevent the authors from making accurate conclusions about feeding (see also next point). First, for many genotypes the authors do not actually measure this phenotype. Instead, the authors rely heavily on developmental delays and body size to make conclusions about feeding (THD’>Kir2.1;THD’>NaChBac;C929-GAL4>Dop1R1; MNSC ilp3 overexpression and knockdown; ilp3 knockdown in STIMKO). This is not appropriate; while feeding will certainly affect body size and larval development, many things that affect larval development and body size have no effect on feeding. In the revised manuscript these genotypes must have accompanying feeding behaviour (and mouth hook contraction data see below) to support a role in feeding.

*Ans: In the revised version we have measured either larval size or adult body weight or both in all key genotypes. These include Fig1E, Fig 3C and 3D, Fig 6F, 6J and 6M, Fig 7C, Suppl Fig 2B, Suppl Fig 5A. In addition we have now performed the mouth hook contraction assay for feeding behaviour of key genotypes. This is quantified and shown in Fig 1K, Fig 2I, Fig 3A, Fig 6N and videos 1-5.*

3. Second, and the assays used to monitor feeding are not ideal. A much better and easier assay is to count mouth hook contractions across 15-20 animals for 30 sec per animal. This will tell you whether the animal is motivated and trying to feed independently of how much food it can hold in its gut. Another factor that may complicate the feeding phenotype is whether the larvae have initiated wandering prematurely. This can be easily monitored by asking where the animals are found (are they in the food still but not eating or are they leaving the food where there is none to consume?). These tests will help the authors make more accurate conclusions about the phenotype of the STIMKO larvae is caused by precocious wandering (as described in Britton et al 2002) or reduced feeding.

*Ans: Mouth hook contraction data are now shown in Figs 1K, Fig 2I, Fig 3A and Fig 6N and in Videos 1-4. We have measured mouth hook contractions from 12-15 animals per genotype. From our observations STIM^KO^ larvae were never found to leave the food indicating that they do not undergo precocious wandering.*

4. RNAseq data of STIMKO and CS brains must be presented for evaluation by reviewers and readers. There may be large trends that are visible when an unbiased analysis of the data is presented. Unfortunately this was not made available.

*Ans: We have deposited the data in a publicly available database and a link to these data is given in line 384.*

5. There must be connections made between the dopamine receptor in MNSC and the insulin responses in STIMKO animals to fully support the authors’ model that dopamine from THD’ neurons mediates the changes in ilp levels in MNSC. The authors must determine whether phenotypes caused by loss of Dop1R1 in THD’ neurons (including feeding and developmental) are rescued by overexpression/knockdown of ilp levels.

*Ans: We thank the reviewer for these insightful comments. To address these we have included data from several new experiments. In the revised manuscript we show knockdown of the dopamine synthesizing enzyme TH in THD’ neurons affects BOTH feeding and growth (Fig 3A-D). Further we show that Dop1R1 is required on MNSc for larval development and growth (Fig 7A-C). We also show that MNSc respond to Dopamine (Figure 7D and lines 430-437). ilp3 overexpression affects growth negatively BUT does not change feeding rate (Fig 6K-N). Our current revised model thus support dopamine from THD’ in maintaining feeding independent of the ilps and in regulating larval growth through ilps.*

6. Statistical tests must be adjusted throughout – according to the methods the authors used t-tests. In most cases (any time there are more than two genotypes, which is most experiments, the essential test is a one-way ANOVA using multiple comparisons to obtain p-values. All of the comparisons should be shown on the graphs (not just one comparison that is significant, some comparisons might be not significant or ‘ns’). The authors must determine whether the rescued genotype is significantly different from all control genotypes (see note 1 on correct genotypes).

*Ans: Appropriate statistical tests, t-tests for two genotypes and ANOVA for more than 2 genotypes, have been performed for all experiments. We have indicated significance in the figures, mentioned the appropriate statistical test in the figure legend and added a Supplementary table stating the P values for all relevant genotypes in figures at all key time points.*
Minor problems
1. The reason for the heterogenenous response of THD’>dsSTIM animals’ calcium response to KCl is unclear. Because there are a number of sex differences in the mechanisms underlying larval growth, and in the dopaminergic system, the authors should separate the sexes to determine whether males and females show different responses to loss of STIM in THD’ calcium responses (and other heterogeneous phenotypes).

*Ans: Our experimental developmental time point for measuring the KCl response is the mid 2^nd^ instar larval stage (just prior to when the larvae become visibly sickly). We could not differentiate between male and female larvae at this stage because the gonads are not yet developed. We have now measured the adult weight of THD’>dsSTIM animals and these do not appear different between males and females, though they weigh significantly less than control genotypes (Fig S2C).*

2. The precise genotypes are not always listed in figures or in the text. Complete genotypes should always be available to readers for the final publication in all those locations.

*Ans: We have added complete genotypes, where missing, in the figure legends.*

3. The authors should cite the Britton paper from 2002 in which they show that changes in insulin pathway activity cause the animals to leave the food, as that is a relevant finding to the authors’ potential identification of ilp3 in regulating feeding.

*Ans : Present data show that ilp3 does not regulate feeding (Fig 6M) but affects growth. Hence this paper has not been cited.*

4. Line 55 tachykinin is misspelled.

*Ans: We have now removed the reference to tachykinin.*

5. There are many feeding studies and hormones that affect feeding not cited in the introduction (including but not limited to ecdysone, Akh, etc). Please include a better more detailed introduction to the rich literature on this topic, as there are many studies.

*Ans: Based on comments from the other reviewers we have now restructured the introduction and focused primarily on references related to larval feeding.*

6. Line 74 – I don’t agree with the statement that circuits that maintain feeding are not well understood. I think there is a lot of literature in this space, so the authors should moderate this claim.

*Ans: We have modified this statement.*

7. Line 106 – I don’t agree with the statement that the small size of the STIMKO must be due to a deficit in cell division. Many larval tissues are endoreplicating, so they grow but don’t really divide. Also, there are many nonautonomous effects of specific cell types like the fat body on growth that don’t involve problems in cell division. The authors will need to adjust the text for accuracy.

*Ans: We have modified this statement.*

8. Because the authors show that there are defects in neuroblast cell divisions, they will need to quantify the number of THD’ cells in STIMKO mutants. Currently they say the THD’ cells “appeared no different in STIMKO…” – this claim must be backed up by data given the neuroblasts defects.

*Ans: Number of THD’ cells have been counted and the data is shown in Supplementary figure 3A. Since larval dopaminergic neurons are specified during embryonic neurogenesis, there is no change in the number of THD’ cells (ref 34).*

9. The colors in the images should be changed from red and green to magenta and green to be more accessible to individuals with visual impairments.

*Ans: This change has been made through all the figures.*

10. Proving a physiological role for STIM in THD’ neurons should include evidence that the gene is normally expressed there.

*Ans: We agree but unfortunately the STIM antibody does not work for immunohistochemistry. Our observation that depletion of STIM with an RNAi in THD’ cells reproduces phenotypes similar to the STIM null supports a requirement for STIM in THD’ neurons.*

11. Please state your food recipe, as this is essential information in replicating larval *experiments.*

*Ans: Added*
12. Please reference Delanoue et al 2009 for the pupal volume calculation.

*Ans: Added (ref 80)*

13. Please acknowledge Flybase and its funding, the Bloomington Drosophila stock center and its funding, and VDRC and its funding.

*Ans: Added to acknowledgments (lines 791-792).*

**Have all data underlying the figures and results presented in the manuscript been provided?**
Large-scale datasets should be made available via a public repository as described in the *PLOS Genetics* [data availability policy](http://journals.plos.org/plosgenetics/s/data-availability), and numerical data that underlies graphs or summary statistics should be provided in spreadsheet form as supporting information.

Reviewer #1: Yes

Reviewer #2: Yes

Reviewer #3: **No:** There is RNAseq data that is mentioned as rationale for doing a series of experiments on ilp3 that is never presented or analyzed at all "will be published in future". For publication in an open-access journal, to me this is unacceptable.
