# Supplementary material for: A STIM dependent dopamine-neuropeptide axis maintains the larval drive to feed and grow in Drosophila
Source: PLoS Genet. 2023 Jun 26;19(6):e1010435. doi: 10.1371/journal.pgen.1010435 (PMC10328320; doi:10.1371/journal.pgen.1010435)
Supplement: Attachment — Submitted filename: Response to Reviewers_0506_V3.docx [file pgen.1010435.s018.docx]

Dear Greg,

We have now further revised our manuscript “**A STIM dependent dopamine-neuropeptide axis maintains the larval drive to feed and grow in Drosophila” to address comments from the reviewers. We include a point by point response to comments from both reviewers. Please note that the title has undergone a minor change and now says ‘dopamine-neuropeptide axis’ instead of ‘dopamine-insulin axis’ to address the first specific comment by reviewer 1. References 68 and 69 from the previous version are no longer included because they referred to insulin-dopamine modulation in mammals and reviewer 1 suggested they were misleading.** As asked by reviewer 3 we have now included the RNAseq data and analysis in the revised manuscript. Consequently ref 79 has been added as it refers to a method for the RNA-seq.

Yours Sincerely,

Gaiti Hasan

Response to reviewers:

Reviewer #1: The findings on the STIM mutation, its site of action (a few TH neurons), and its systemic impact are very interesting and intriguing. The additional data on the neuropeptide Ilp3, TH and dopamine receptors are informative.
However, the central question remains unresolved whether THD's dopaminergic neurons regulate larval growth and feeding independently or whether the growth defect is a consequence of feeding, as the authors suggest, and how the two processes may be linked or mediated. This uncertainty persists because silencing the Ilp3 peptide, upregulated in STIM_KO larvae, partially rescues the growth defect and viability without affecting feeding, indicating that these processes may be independent. The finding that Ilp3 is upregulated in STIM-KO also raises questions about whether the growth defect is caused by a nutrient deficiency or the presence of abnormal levels of Ilp3 and/or other factors that arrest the developmental progression and further growth.

Some specific comments:
The authors declare in their rebuttal that Ilp3 does not affect feeding rate and "..... We have appropriately modified our conclusions to reflect these data." However, the revised paper does not consistently update its conclusions throughout the text, and the model is still based on assumptions requiring further work. For instance, the original model and conclusions are still reflected in the title, even though they are not supported by the current data. Likewise, in the authors' summary, the following text: "The identified dopaminergic neurons dysregulate the normal pattern of larval ilp3 expression leading to premature cessation of feeding and growth" it may suggest that Ilp3 mediates both effects, which creates false expectations for readers. In line 103, "We identified a novel dopaminergic-neuropeptide connection that drives persistent feeding and growth in early larvae." The study does not identify the neuropeptide(s) downstream of the dopaminergic neurons associated with persistent feeding, and the role in growth remains incompletely characterized.

*Ans: We have now modified the summary appropriately to reflect the new findings with ilp3 (line 47). Similarly we have gone through the text and made appropriate changes to address this issue (lines 104, 459-461). We have modified the original title to state dopamine-neuropeptide axis.*

Figure 1E shows the measurement of larval length at specific chronological ages. The figure is informative. However, it should be noted that these measurements only reflect the cumulative increase in size over time and should not be confused with growth rate, which is measured differently with a defined formula. Relevantly, given the significant delay of STIM_KO mutants entering L2 and L3 around 20-24 or more hours later than the control, the authors must compare developmental age, not chronological age. With such delay, the chronological age is irrelevant. Based on the authors' carefully collected data on chronologically aged larvae in Figure 1, the control larvae at 58-62 h are in L2, while the STIM_KO larvae only enter the L2 at 72-78 h. Hence, the 72-74 h STIM_KO larvae are of a developmental age approximately that of the control larvae at 58-62 h. Few differences are noticed by comparing the sizes of 58-62 h larvae (control) with the size of 72-74 h (mutant). The mutant larvae appear severely delayed in their development but arrive at approximately the expected size of the L1 and L2. Yet, as observed by the authors, the larvae do not seem to progress or grow further, and larvae size decreases after 84 h.
The mutant larvae already experience a feeding impairment from their early stages of development, which could contribute to their delayed growth. The mutant larvae require additional time, but they seem to reach the appropriate size for moulting. Once feeding ceases, the larvae become arrested and cannot continue growing, and the water loss may explain the decline in body weight or length.
In this case, comparing the length of larvae between animals of the same chronological age is inaccurate. By using this method, the authors may come to incorrect conclusions.

*Ans: We agree that comparisons by chronological age of STIM^KO^ larvae with controls could be misleading. Control larvae at 60h are equivalent to STIM^KO^ at 72h. We have been careful not to make a direct comparison between developmental ages of control and STIM^KO^ larvae). This is evident in Figure 1D and the quantification of larval length in Fig 1E (lines 118-126). At earlier timepoints also STIM^KO^ larvae are significantly smaller then controls starting from the first time point measured (34-38h, Fig 1E) indicating slower growth of STIM^KO^ between 34-38h and 70-74h. To avoid misinterpretations based on chronological age all cellular and molecular comparisons between control L2 and STIM^KO^ L2 were performed with controls at 58-62 hrs and STIM^KO^ at 70-74h (Fig 3, 4 and 6).*

The role of Ilp3 is important for understanding STIM defects. The authors suggest that Ilp3 acts as an antigrowth signal based on animals' larger and smaller sizes when Ilp3 is manipulated. This idea counters the Ilp3-KO mutant defect. "...mutants lacking Dilp3 develop with normal timing and show
only a modest reduction in fecundity" (https://doi.org/10.1038/ncomms7846). An analysis using endogenous Ilp3
mutations must clarify this point. Moreover, an increase in size may result from an increase in growth rate or a longer growth time at a normal or reduced growth rate. Therefore, caution must be exercised without proper analysis since there are numerous examples of mutants with larger pupae and adults due to developmental extension and delay without such factors being an antigrowth signal. In the case of dsIlp3 animals, the significant delay in their development may explain the increase in the size of their larvae and pupae, either in part or in whole, through an indirect effect on size due to delayed maturation time. Overexpression of Ilp3 also leads to developmental arrest and delay. If Ilp3 inhibits growth, the growth rate should increase relative to developmentally age-matched controls, and developmental time should be accelerated rather than delayed —as seen in SDR mutations with clear increases in growth rate. However, if growth is not the primary effect, larval growth is not expected to increase above age-matched controls. To affirm the antigrowth action of Ilp3, the authors must measure larval growth rate over time and compare it properly.

Starved animals (which grow slowly or do not grow) decrease rather than increase Ilp3 transcription (Ikeya et al., 2002) and increase other ILPs such as ILP6. If the authors are correct, STIM-deficient larvae with reduced feeding should show signs of starvation. The authors' data on Ilp genes presented in the article do not support with Ilp3 increased and ILP6 unchanged. The upregulation of Ilp3 transcription, despite being on slow feeding, is intriguing and does not add well to the model proposed by the authors. The model seems too simplistic.

*Ans: Our conclusion that ilp3 functions as an antigrowth signal in L2 and early L3 larvae derives from the data in Fig 6H-M, where overexpression of ilp3 leads to smaller sized larvae and knockdown leads to larger sized larvae. Smaller larvae upon overexpression of ilp3 support a role for ilp3 that is antigrowth. It is possible that the increase in size is due to the reasons suggested by the reviewer. The previous study with ilp3 null mutants (*[*https://doi.org/10.1038/ncomms7846*](https://doi.org/10.1038/ncomms7846)*) cannot be compared directly with our study. Normal growth and fecundity of ilp3 mutants was observed in yeast-enriched media. On normal media (similar to our growth conditions) ilp3 mutants exhibit slower pupariation. Similarly, the study by Ikeya et al (2002) looks at ilp3 transcripts upon starvation in mid L3 (96h) whereas we observe upregulation of ilp3 in STIM^KO^ larvae in mid-L2. Given the dynamic nature of ilp3 transcription through larval development (Fig 6C) the two sets of data cannot be compared directly. We agree that the ilp3 phenotypes and function need further investigation and this will be pursued in future.*

This reviewer suggested L-DOPA supplementation experiments clarify the role of dopamine downstream of THD' STIM-KO. Still, the authors justify not doing the experiment by arguing that the mutants are defective in feeding and would not work. Surprisingly, their Discussion describes that such an experiment works in mice with a feeding defect due to a lack of dopamine. Lines 506-509 "where prenatal mice genetically deficient for dopamine (DA-/-) could not feed and died from starvation. Feeding could, however, be initiated upon supplementation with L-DOPA (64), allowing them to survive. "

*Ans: The experiment in mice involved either forced feeding OR injections of L-Dopa. This is technically challenging in fly larvae. We have mentioned this in the discussion (line 518).*

The experiments with TH-RNAi and dopamine receptors in limited neurons are informative but insufficient. The TH-RNAi defect recapitulates some aspects of the STIM defect. Still, it does not mimic the effects of STIM deficiency, which is lethal, so there is more than suppressing the dopaminergic pathway. L-DOPA supplementation could clarify the impact of impaired feeding and what are causes and consequences. The observation that none of the manipulations causes the lethality observed in STIM deficiency cannot be solely explained by RNAi not being a complete knockout because the lethality is seen in STIM-RNAi. The STIM defect is more complex than the model suggests.

*Ans: We have included a sentence regarding the phenotype of STIM^KO^ larvae and how it likely derives from the THD’ cells and other cells (lines 192-195).*

Furthermore, the authors propose, "In summary, we have identified a novel STIM-dependent dopamine-ILP circuit that regulates developmental changes in larval feeding behaviour"; however, such as instructive role is an overinterpretation. An instructive role in coordinating developmental feeding behaviour (e.g. rate of feeding and stop feeding) must be demonstrated by showing that STIM can drive larvae to eat when they stop eating (at wandering) or feed faster and grow faster. The authors are also mistaken in describing that wandering larvae feeding stops gradually. Feeding slows gradually after 96 h and stops entirely as larvae wander out of the food to pupate.

*Ans: As mentioned in the introduction the role of THD’ cells in larval feeding has been investigated in second instar and early third instar larvae (line 99-100) where expression of STIM^+^ rescues feeding deficits (Fig 2I). Studies in late third instar larvae would be of interest and will be pursued. We have corrected the statement regarding feeding by wandering larvae (line 96).*

Minor issues:
• Figure 4 shows that the dendrites are labelled with anti-RFP. This antibody is not included in the Methods. Although mCherry is a red fluorescent protein, describing the DenMark (mCherry) construct in the Resource Table is more appropriate (see flyBase and ref .40)

*Ans: We have now included the anti-RFP antibody in the methods (Lines 685-686). The resource table has been modified to state that flies with the DenMark construct contain mCherry.*

• Syt-eGFP

*Ans: This has been corrected in the text and figures.*

• MNSc is described in different ways, including "MNC"; the original description was mNSC.

*Ans: we have corrected MNSc throughout the text and figures.*

• "Quantification of the weight of 10 flies from the indicated genotypes". The sex of the flies must be indicated.

*Ans: All weights are of 5 females and 5 males. This is mentioned in the methods (line 664).*

• The authors state, "synthesis and release of the steroid hormone 20-OH ecdysone from the prothoracic gland … (16,17). This statement is incorrect. The prothoracic gland releases pro-ecdysone (alpha-ecdysone), converted peripherally to 20-OH ecdysone and other ecdysteroids.

*Ans: We have corrected this statement (line 90).*

• The Discussion pertains to juvenile animals, but the references to the insulin-dopaminergic axis in other species relate to adult animals. This must be explicitly stated in the text or compared to a parallel system involving juvenile animals or children. References 68 and 69 predominantly concern the role of insulin in dopamine modulation, so the. A suggestion of conservation between the two is misleading.

*Ans: We have specified which stage each study refers to in the discussion. As suggested references 68 and 69 have been removed.*

In summary, authors should review their manuscript and ensure consistency between their conclusions and presented data, interpreting the data correctly and without overinterpretation or assumptions regarding mutant behaviour without supporting data. This is important because the previous version had some unsupported assumptions, and therefore the description of the phenotype should be moderated throughout the manuscript according to the available data. The phenotype and data remain intriguing, but it is unclear whether it is simply a feeding defect that becomes chronic and causes other alterations, leading to growth arrest and death, or if the mutation additionally affects systemic processes that influence growth along with persistent feeding. The role of the THD neurons and dopamine in normal development and changes in growth patterns throughout development is unclear.

Reviewer #3: I thank the authors for their efforts to address my concerns. While the authors have addressed several concerns in part, they have not fully addressed them.

Major comments

1. Following up on my point in the original review about using the correct genotypes in doing a rescue experiment. In their response to reviewers the authors state they used the genotypes I set out in my original review in the bar graph in Figure S2A, and have “performed the appropriate genetic controls for all other genotypes tested and have included them either in the main figure or the associated supplementary figure.”

Perhaps this information was missing somehow from the figures but I can only find the appropriate genotypes that must be used when doing all rescue experiments in Figure S2A. As far as I can tell from the material to which I have access, the rescue experiments simply use Canton S, the StimKO, and then the rescue genotype. These are not the correct control genotypes for the reasons I set out in my original review.

At this point, the authors will need to moderate their conclusions in these other experiments by saying “as far as we can tell from the control genotypes we used, there was a rescue; however, future experiments will need to verify this conclusion by showing the UAS and GAL4 lines alone do not provide any significant rescue in this phenotype”.

*Ans: As suggested we have suitably modified this statement (lines 201-206).*

2. I am still unsure why the RNAseq data analysis was not shown and why this technique and analysis workflow was not included in the methods. For readers and reviewers to evaluate this data the workflow must be presented and the data shown including a statistical analysis.

*Ans: We have added a Supplementary figure (S5A Fig) and a Supplementary Table (S3 Table) with the RNAseq data and its statistical analysis. A section describing the RNAseq methods has been included in the methods section.*

3. In Line 449 the authors draw a direct line between the THD’ neurons, the feeding deficit, and the MNSCs – their feeding data with dilp3 contradicts this sentence and it will need to be modified.

*Ans: This has been corrected.*
